# Oral health in the context of prevention of absenteeism and presenteeism in the workplace

**DOI:** 10.5327/Z1679443520190397

**Published:** 2019-12-01

**Authors:** Roberto Brasil Lima, Alexander Buarque

**Affiliations:** 1 Specialization in Occupational Medicine, Universidade de São Paulo - São Paulo (SP), Brazil. Universidade de São Paulo Specialization in Occupational Medicine Universidade de São Paulo Brazil

**Keywords:** oral health, absenteeism, presenteeism, occupational dentistry, occupational health

## Abstract

**Background:**

Oral health is an important factor of human morbidity worldwide. Yet is often neglected in occupational health despite its direct impact on the quality of life and health of workers.

**Objective:**

To discuss the role of oral health in sickness absence and presenteeism, as well as in development and work efficiency improvement processes involving governments, companies and the workers themselves.

**Methods:**

Review of full-text articles on oral health, occupational health, sickness absence and presenteeism published in English or Portuguese in the last 10 years and included in scientific databases.

**Results:**

Oral problems accounted for 9 to 27% cases of sickness absence and 28 to 50% of presenteeism, with toothache and temporomandibular joint pain as the most frequent reasons. About 50% of workers prefer company-provided dental care, while 40% visit public and 10% other types of facilities. Despite high, the prevalence of oral diseases and orofacial pain was not associated with high rates of absenteeism, but mainly with presenteeism, this is to say, workers do not tend to miss work days, but their performance is reduced and become susceptible to more serious health problems in the future.

**Conclusion:**

Oral health is not dissociated from general and occupational health, and as such it must be enhanced and duly promoted in an integrated manner. Effective and comprehensive oral health promotion and prevention public policies and private sector actions in the workplace can enhance the quality of life of workers.

## RESUMO |

A saúde bucal é um importante fator de morbidade humana mundial, mas frequentemente negligenciada em saúde ocupacional, apesar do impacto direto na qualidade de vida e na saúde do trabalhador. O objetivo deste artigo foi discutir o papel da saúde bucal como fator de absenteísmo e presenteísmo e nos processos de desenvolvimento e eficiência laboral por parte de governos, empresas e pelos próprios trabalhadores. Realizou-se revisão da literatura com artigos na íntegra, em inglês ou português, dos últimos 10 anos, nas bases científicas relacionando-se os temas saúde bucal, saúde ocupacional, absenteísmo e presenteísmo. Doenças bucais variaram como causas de absenteísmo entre 9 e 27%, mas de 28 a 50% como de presenteísmo. Dores dentárias e de ATM são as causas mais comuns. Entre 9 e 56% dos trabalhadores buscaram tratamentos em rede pública, e de 40 a 45%, em rede privada. Nas empresas que oferecem atendimento odontológico, 49% dos funcionários optaram pelo ambulatório da empresa, contra 40% da rede pública e 11% de outros tipos. A elevada prevalência de doenças bucais e de dor orofacial não é causa de elevados índices de absenteísmo, mas principalmente de presenteísmo. O trabalhador não se afasta, trabalha de forma deficitária e com propensão a maiores adoecimentos futuros. A saúde bucal tem importância não dissociada da saúde geral e ocupacional e, como tal, precisa ser incentivada e acompanhada de forma integrada. Políticas públicas e ações do setor privado efetivas e abrangentes na promoção e prevenção em saúde bucal no ambiente laboral podem ofertar melhor qualidade de vida aos trabalhadores.

## INTRODUCTION

Pain has considerable impact on human life as a function of the suffering it causes and the limitations it brings into activities of daily living, in addition to dramatic effects on society at large as a function of the high costs of treatment and productivity loss^1^. Pain impairs sleep, leisure and interpersonal relationships. From the occupational perspective, it is a cause of absenteeism and presenteeism, or type 1 and 2 absenteeism as they are also known^2^^,^^3^.

Musculoskeletal and psycho-emotional disorders are significant causes of illness and the leading reasons for absenteeism worldwide^4^. Oral health, in turn, is scarcely addressed and often neglected within occupational health, even though it is a considerable global cause of disease. According to the World Health Organization (WHO) oral disorders may cause pain, distress, embarrassment and social deprivation, resulting in individual and collective harms^1^. Studies which analyzed the relationship between oral health and quality of life indicate that pain is common among individuals with poor oral health and has immediate impact on their quality of life^5^.

Several workers depend on the mouth to perform their job, as e.g. musicians, actors, models, journalists, teachers, salespeople, enologists, bartenders, chefs and cooks, among others. In turn, any work environment exposes workers to several potentially harmful agents with considerable relevance for the oral cavity structures^6^. As a part of the digestive system, the oral cavity is related to functions such as absorption, retention and excretion and is subjected to physical, biological, chemical, ergonomic and traumatic hazards which may directly interfere with the performance and productivity of workers^7^.

Given the relevance of the oral cavity for human health and a direct relationship between oral and occupational health, understanding ongoing perceptions of governments, organizations and the workers themselves about the relationship between these factors and job performance, productivity and efficiency and their impact on the quality of life of workers is highly relevant. Therefore, the aims of the present study were to evidence, based on a literature review, the role of oral health as a cause of sickness absence and presenteeism and to analyze current interventions within occupational health.

## METHODS

We performed a narrative review of full-text articles published in English or Portuguese in the past ten years and included in databases Scientific Library Online (SciELO), MEDLINE, PubMed and Cochrane using keywords: occupational dentistry, oral health, workers’ health, absenteeism and presenteeism, and the equivalent terms in Portuguese. We included studies with focus on oral and occupational health, absenteeism and presenteeism, being systematic reviews, meta-analyses, cohort, case-control or cross-sectional studies. Studies which addressed occupational or oral health alone were excluded.

## LITERATURE REVIEW

Work began together with the first human being on Earth, however, the relevance of studying the health-disease process within the work environment has been acknowledged rather recently^8^^,^^9^. Hippocrates (460-375 BC) made the first mentions to occupational diseases in the book *On Airs, Waters and Places*, in which he described a clinical case of lead poisoning, however, without any reference to the patient’s workplace or occupation. These topics became relevant only after Bernardino Ramazzini’s studies, published in his book *De morbis artificum diatriba*, from 1700, in which he discussed diseases related to 54 occupational categories. This work also contains the first mentions to the relationship between oral disorders and workplace^10^.

The Industrial Revolution (19th century) called the attention to and brought the first attempts at implementing occupational health actions as a result of a vindication movement launched by English workers in the face of inhuman exploitation of the workforce for the sake of profit and development. As a result, the Health and Morals of Apprentices Act was passed in 1802, although it was never effectively enforced due to lack of the due inspections. Therefore, a Factory Act was passed in 1833, which established an autarchic government agency for the surveillance of occupational health in factories. Finally, in 1957 WHO and the International Labor Organization set guidelines, which are still in force and are periodically updated, to raise awareness on and survey occupational health^11^.

Several authors reported a relationship among occupational activity, sickness absence and oral diseases^9^. In 1970 Medeiros and Bijella^12^ commented that one of the earliest records on dental care for workers corresponds to the English railway system, with the establishment of dental offices in companies starting 1915. Legge^13^ observed in 1937 that dental care provided by dentists soon found a place in the health programs of different organizations. In 1947, Goldhorn emphasized the relevance of dental care in industrial health programs^14^, and in 1957 Salzmann discussed the role of dentists in the industrial setting, particularly of those with training on the prevention, diagnosis and treatment of oral manifestations of occupational diseases^15^. From a more modern and multidisciplinary occupational health perspective, several oral disorders might be related to work, which points to the relevance of dental care in the workplace as a means to reduce illness and absenteeism^7^.

Research on absenteeism has broad repercussions for different populations, fields and even at the national level, because it enables analyzing causal factors that link work to the health profile of workers, as well as planning prevention actions to improve productivity and the state of health of all workers^16^^,^^17^^,^^18^. Workers are exposed to the same conditions which cause disease among the overall population, to which workplace environmental factors should be added-as e.g. hazards inherent to each job and working conditions-which may make the situation worse and increase the risk of harm to human health. Older age, poorer educational level, increased exposure to occupational hazards and unhealthy habits such as smoking and substance abuse, among other factors, are associated with higher incidence of absenteeism^16^^,^^19^. In the United States, several studies found that oral health problems were associated with 0.25 to 1.5 lost working hours/population/year and a loss in productivity of more than USD 800 million^20^^,^^21^. Similarly, estimates for the United Kingdom indicate that 415,000 people/year lose one full working day due to oral health problems, with an average daily cost of more than £80 and an annual productivity loss equivalent to £36 million^22^.

Term absenteeism designates an employee’s absence from work due to disease and is easily measured together with its cost in terms of lost productivity. In turn, presenteeism, or type 2 absenteeism, represents the situation in which sick workers do not miss work days, but are unable to achieve their best performance, with the consequent loss of productivity^23^. Presenteeism includes aspects related to health, personality, job and occupation^24^. Self-perceived illness, older age, female sex, sociocultural factors and job opportunities, demands and characteristics are aspects associated with presenteeism^25^^,^^26^. This phenomenon might have even poorer effects on the health of workers and their coworkers, because it triggers additional comorbidities and predisposes to future illness^27^. In turn, its associated costs are higher than those of absenteeism, because presenteeism impairs individual productivity over a long period of time^28^.

## RESULTS

Based on the preset inclusion and exclusion criteria we selected 11 studies for analysis (Table 1). Since the number of records was too small, we did not perform quantitative analysis.

**Table 1.** Summary of selected studies.

Author/year	Aims	Methods	Conclusions
Hayes et al., 2013^29^	To quantify time loss due to dental problems and treatment in the Canadian population, to identify factors associated with this time loss and to provide information on the economic impacts of these issues	Data from the 2007/09 Canadian Health Measures Survey System. Descriptive analysis determined the proportion of those surveyed who reported time loss and the mean hours lost. Linear and logistic regressions were employed to determine what factors predicted hours lost and reporting time loss respectively. Productivity losses were estimated using the lost wages approach	Over 40 million hours per year are potentially lost annually due to dental problems and treatment in Canada, with subsequent potential productivity losses of over 1 billion dollars. These losses are comparable to those experienced for other illnesses (e.g. musculoskeletal sprains). Further investigation into the underlying reasons for time loss and which aspects of daily living are impacted by this time loss are necessary for a fuller understanding of the policy implications associated with the economic impacts of dental problems and treatment in Canadian society
Harford and Chrisopoulos, 2012^30^	To estimate the cost of lost productivity associated with oral problems and to examine whether any demographic, oral health or dental visiting characteristics are associated with lost productivity	Data sourced from the National Dental Telephone Interview Survey (NDTIS) 2010 corresponding to a sample of 6,284 citizens aged 18 and over	The estimated loss of productivity among Australian workers combining the cost of time loss and dental visits was 808 million dollars
Capelari et al., 2015^31^	To analyze characteristics and causes of absenteeism for dental reasons based on International Classification of Diseases, 10th revision (ICD-10) codes indicated in certificates granted to municipal civil servants in the interior of the state of São Paulo, Brazil	Cross-sectional study performed in Santa Cruz do Rio Pardo, involving analysis of records of civil servants relative to the period from 2001 to 2012; 343 records were randomly selected. Causes of sickness absence for dental reasons were quantified based on ICD-10 codes	The high rate of sickness absence found points to the need for occupational health promotion and prevention policies, with emphasis on dental care as a means to reduce absenteeism
Barros et al., 2014^32^	To calculate the rate of absenteeism due to medical or dental reasons and analyze their correlation with sociodemographic and occupational variables	The authors analyzed 387 medical records of employees of a graphic design and information company along 24 months and investigated correlations between medical certificates and sociodemographic (age, sex) and occupational (time in the job, occupational category) variables to characterize absenteeism and presenteeism	Absenteeism for medical reasons was more frequent than sickness absence for dental problems. Nevertheless, the latter was the 11th most frequent cause of sick leave. Oral health problems limit the workers’ performance, which may be measured based on absenteeism and presenteeism rates
Nardi et al., 2009^33^	To investigate the relationship between orofacial pain and sickness absence among slaughter and meat processing industry workers in the South region of Brazil	Cross-sectional study with 401 slaughter and meat processing industry workers in the South region of Brazil. A self-reported questionnaire included 9 different types of pain leading to sickness absence and duration of sick leave	The prevalence of sickness absence due to orofacial pain was low
Lacerda et al, 2011^34^	To establish the prevalence of orofacial pain and its impact on the performance of textile industry workers in Laguna, Brazil	Cross-sectional study performed in 2004 with all 267 employees of 5 textile industry companies. Data were collected through administration of Locker and Gruska’s questionnaire to measure the impact of oral health on daily performance	The prevalence of orofacial pain was high and represented the main determinant of the impact of poor oral health on daily performance at work
Miotto et al., 2012^35^	To establish the prevalence of toothache and sickness absence due to toothache and its association with sociodemographic variables	Cross-sectional study with a random sample of 170 of 545 civil servants at the municipal government of Venda Nova do Imigrante, Espírito Santo, Brazil. Data collection was performed by means of a 27-item questionnaire administered by one municipal civil servant	The prevalence of sickness absence due to toothache was higher among males, employees with low educational level (incomplete secondary school) and family income up to twice the equivalent of the minimum wage. The results point to the need for oral health promotion policies to improve the quality of life of workers
Lacerda et al., 2008^36^	To establish the prevalence orofacial pain and its association with sickness absence among metallurgy workers in Xanxerê, Santa Catarina, Brazil	Cross-sectional study with all 480 male employees of 13 metallurgy companies. Participants were subjected to structured interviews. Data were also collected from the human resources departments	The prevalence of orofacial pain was high
Resende et al., 2009^37^	To identify the leading causes of sickness absence due to dental reasons among employees of an electricity distribution company	Cross-sectional study. Data were collected by means of a questionnaire delivered to the health care manager	The highest rate of sickness absence corresponded to male workers, aged 41 to 50, having completed secondary school and allocated to the production department. Dental certificates should be analyzed by dentists
Miotto et al., 2013^38^	To establish the prevalence of toothache and sickness absence and their association with sociodemographic variables	Cross-sectional study with a random sample of 169 of 666 workers. Data were collected by means of a structured questionnaire	The prevalence of pain was considerable, but was not associated with any of the analyzed variables. Pain was a reason for absenteeism, which was associated with the participants’ educational level. Periodic oral health examinations should be incentivized to enable early diagnosis and intervention and thus minimize toothache episodes
Miotto et al., 2014^39^	To establish the prevalence of toothache and sickness absence and their association with sociodemographic variables	Cross-sectional study with a random sample of 312 out of 994 civil servants at the municipal government of Marataízes, Espírito Santo, Brazil. A 27-item questionnaire was administered by three trained interviewers	The prevalence of toothache was high and was a reason for sickness absence, especially among workers from the lower economic classes and with poorer educational level

As concerns economic impacts, Hayes et al.^29^ reported in 2013 that over 40 million hours/year were lost due to dental problems and treatment, corresponding to a mean of 3.5 lost hours per person and productivity losses of over 1 billion dollars. Following a nationwide survey in Australia, Harford and Chrisopoulos found a loss of 1.56 hours/year/person to dental problems and productivity losses of over 800 million dollars.

According to several studies, the rate of absenteeism due to oral problems ranges from 9.06% to 26.7%^30^^,^^31^. However, a survey of medical certificates granting leaves longer than 15 days for dental reasons indicates that the actual rate is rather low, representing less than 5% of all medical certificates^32^^,^^33^. In turn, 28.5% to 50% of workers do go to work when with toothache^34^^,^^35^, which situation impairs their productivity as a function of loss of attention, irritability, anxiety, depression and also work accidents.

Some data in the literature are conflicting. The influence of sex varies according to the analyzed population or is insignificant. Similarly, there are reports indicating that absenteeism is more frequent among workers with low educational level, i.e. incomplete secondary school or less, those who attended higher education, or again, is irrelevant. The same is the case of age. In turn, the prevalence of absenteeism due to orofacial pain exhibits a more consistent correlation with lower family income (up to three times the equivalent of the minimum wage) and more years in the job^31^^,^^35^^,^^36^^,^^37^^,^^38^^,^^39^.

The orofacial problems most frequently reported by workers include spontaneous toothache or caused by cold or warm fluids or sweets, temporomandibular joint (TMJ) pain, pain upon opening the mouth or chewing, pain in the eye or in the anterior part of the ear and feelings of burning in the cheeks and tongue^31^^,^^32^^,^^36^^,^^38^. Spontaneous toothache or caused by pressure or temperature, TMJ pain and pain upon chewing are the most common, with rates varying from 32.2 to 66.1%^34^^,^^36^.

According to authors who analyzed treatments for oral conditions, the rate of workers who seek dental care ranges from 21 to 62%^24^^,^^25^^,^^26^^,^^27^^,^^28^^,^^29^^,^^30^^,^^31^^,^^32^^,^^33^^,^^34^^,^^35^^,^^36^^,^^38^^,^^39^, 9 to 56% in public and 40 to 45% in private services^36^^,^^38^^,^^39^. Few studies reported availability of dental care in the workplace, and those which did found that 49% of employees chose employer’s facilities versus 40% who have resource to the public health system and 11% to other services^38^.

## DISCUSSION

Most of the analyzed studies collected data by means of questionnaires, interviews or in databases. While we could not locate any longitudinal study, a large part had cross-sectional design. We found considerable differences in the methods applied, follow-up lasting from 1 month (cross-sectional studies) to 11 years (retrospective studies). Some authors compared sick leave due to medical or dental reasons, others analyzed clinical oral parameters or dental care received, but the vast majority exclusively addressed sick leave due to dental reasons in local organizations. The analyzed populations are heterogeneous in sociodemographic terms, including age, sex, cultural factors, socioeconomic and educational level.

There are few qualitative studies on our subject of interest, which fact might be accounted for by several reasons. For corporate ethical reasons, health is a strategic management subject and thus is considered in terms of investment (when foreseen) or of losses (when unplanned) from the perspective of the relationship between health and productivity, which often hinders the divulgation of the corresponding information. As a result, most publications have no direct link to data sources, but the vast majority are quantitative or consist in literature reviews performed by university-based researchers and restricted to a partial analysis of the problem in terms of magnitude and time.

The last comprehensive survey of oral health in Brazil was performed in 2010^40^. Analysis was stratified per age range (15-19, 35-44 and 65-74 years old) and geographical region. The results evidenced an overall improvement of the oral health of Brazilians by comparison to the situation in 2003. However, the prevalence of oral infectious diseases, tooth loss and malocclusion is still high. The rate of economically active population dissatisfied with their oral health was 25 to 30%, that of oral conditions which interfere with school or work 4 to 13%, and the prevalence of toothache in the past 6 months 9 to 30%.

Dissatisfaction, or self-perceived poor oral health, directly influences the future occurrence of illness, the search for treatment and productivity, results in low self-esteem, and is strongly associated with presenteeism^26^^,^^33^. Pain upon chewing, cleaning the teeth or sleeping is frequently described as having negative impact on well-being, activities of daily living and work. Tooth loss, improper or maladjusted dentures cause dissatisfaction with one’s personal appearance and thus reduce social contacts with other people. These factors might increase more than 22 times the odds for people with orofacial pain to report negative impacts on their daily performance^34^.

The prevalence of absenteeism for dental causes we found, 9% to 27%, is considerable, but the high rate of presenteeism associated to orofacial pain is even more noteworthy, since 28% to 50% of employees go to work with pain or their daily performance is impaired^27^^,^^41^^,^^42^^,^^43^. This finding-and the lower incidence of sick leave due to dental versus other reasons-might be explained by the relatively low intensity and chronic nature of pain, which is usually mild to moderate, and fear of salary deductions due to reduced productivity or of losing the job. At the same time this behavior increases the odds of psycho-emotional disorders, such as depression and irritability, family problems or with coworkers, as well as the risk of work accidents due to loss of attention^21^^,^^24^.

Not all certificates granting sick leave are presented to employers^44^. In addition to underreporting related to compensatory time off, some employees-especially those in higher positions who do not have to account for missing days-do not report absences due to medical or dental reasons. Then, treatment (both scheduled or unplanned) tends to be performed during off-work hours as a result of the corporate culture belief (promoted by human resources departments, and even Safety Engineering and Occupational Medicine Specialized Services-SEOMSS) that producing a dentist’s certificate does not prevent possible payroll deductions, this against the Laws no. 5081/1966 and 6215/1975. In times of high unemployment rates, workers tend to avoid justifying absences by producing health certificates-more particularly for oral health problems-not to give rise to prejudice among coworkers and supervisors, which results in underreporting^45^. Underreporting hinders the planning of occupational health prevention and health promotion actions, distort the results of epidemiological studies and interfere with the attempts at quantifying economic losses associated with absenteeism and presenteeism^46^.

The severity of orofacial pain varies as a function of differences in the pain perception threshold, age, sex, ethnicity, socioeconomic and cultural factors. A low educational level combined with disregard for oral health care and impaired access to dental care contributes to increase the frequency and duration of episodes of orofacial pain^47^. Sickness absence due to oral health problems is usually short and is mostly due to acute pain or following a surgical procedure^33^^,^^46^. Tooth extraction, often resulting from advanced tooth decay or periodontal disease, is a frequent reason for sickness absence^48^. Tooth loss is one of the leading causes of malocclusion and TMJ dysfunction, which in turn are the main reasons reported by workers to miss work days due to orofacial pain. This chain of causality clearly includes also socioeconomic factors, such as educational level, disregard for oral health, access to information and appropriate dental care, in addition to the significant contribution of public and organizational health promotion and prevention policies to improve the quality of life of workers^48^.

Pre-employment oral health examinations are seen as a prevention method helpful to avoid future problems, as it enables diagnosis of oral cavity diseases before individuals enter the workforce. They further contribute to reduce absenteeism and work accidents, as well to epidemiological studies of and impacts on the quality of life of workers^49^^,^^50^. However, in a systematic review performed in 2016 Schaafsma et al.^51^ failed to find evidence for the effectiveness of this approach for any health condition.

The Brazilian Regulatory Standard no. 7 (RS 7) includes a list of chemicals likely to cause oral manifestations of intoxication, as e.g. lead, manganese and mercury. Workers exposed to these chemicals should be periodically subjected to dental examinations^7^^,^^52^. Jobs involving high levels of stress and attention or with long or indefinite working hours (as is the case, e.g. of self-employed workers) are associated with higher frequency of oral health problems^48^. Within this context, periodical oral health examinations might be relevant for early diagnosis and treatment, and thus to reduce the rates of absenteeism and presenteeism. As a function of the degree of risk and type of job, workers should undergo examinations annually or more often^48^. These considerations notwithstanding, RS 7 does not make dental examinations mandatory, but medical coordinators of SEOMSS are enabled to require them as per need (art. 7.3.2 b). Proposals were presented to the Brazilian National Congress to include dental examinations as part of standard occupational health care. Examples are the Law Project no. 3520/2004^53^, which recommends making the inclusion of dentists in SEOMSS mandatory, and Law Project no. 422/2007^54^, which suggests making occupational oral health examinations mandatory. Unfortunately, both projects are still under discussion and have not advanced much. Their overall intention is for employers not to see oral health care as an additional burden, since the focus of these initiatives is not on treatment, but on health promotion and prevention to thus help maintain the productivity levels.

Many workers with orofacial pain who seek care-mostly in public facilities, because private care is expensive-fail to find a satisfactory solution^38^. Long wait times, poor effectiveness, lack and precarious maintenance of equipment are common occurrences in the public sector. Workers who require a large number of visits to dental services tend to miss more work days, probably because the operating hours of the former coincide with the working time of the latter^35^. In their study from 2013, Miotto et al.^38^ found that 49% of workers with orofacial pain sought care at company-based facilities versus 40% at public services and 11% through other means. Ahlberg et al.^55^^,^^56^, in a study from 1996, reported the positive impact of employer-provided dental care on the productivity and well-being of workers by comparison to other approaches. Upon assessing these aspects in Campinas, São Paulo, Brazil, in 2010, Lido and Queluz^57^ found that dental care facilities were more frequently available at large companies, 30% versus 10% among medium-sized businesses, and that pre-employment and periodic oral health examinations were exclusively performed in the former, but only in less than 30% of them. These authors further reported that all the analyzed companies provided dental care to employees, self-managed in 11.11% and through outsourced management in 88.89%.

Companies may deduct dental care provision from their income tax. Outsourced care prevails among the companies which provide this service^57^^,^^58^ mainly through cooperatives or HMOs. Reasons are lack of financial resources, difficulty to manage services and the need to keep the focus on the company’s goals and activities. In 2008 Costa Filho et al.^59^ analyzed the impact of two strategies for dental care provision on organizational costs, to wit, a fixed fee agreement with a HMO and establishing an evidence-based, health-promotion service on the company’s premises (self-management). Both strategies succeeded in the reducing the company’s expenses, 30% and about 50% respectively. However, with the former the number of procedures decreased without any change in their nature, while with the latter also the profile of visits changed, since the number of health promotion procedures increased at the expense of the surgical or restorative ones. In 2002, Pizzatto^60^ observed that availability of dental care in the workplace facilitates access to employees, reduces the time away from the production line and fosters integration among workplace health and safety professionals, while knowledge of the work routine and production process helps dentists to establish a more accurate diagnosis and health promotion and prevention actions better adjusted to the local conditions. In a study from 2011 with 15,338 workers, Oshikohji et al.^61^ found that the rates of oral diseases and tooth loss were lower among those who had received systematic oral primary health care in the workplace, including orientation on hygiene and periodical consultations, by comparison to the employees who had received only eventual or no care at all. While some authors reported that oral health prevention programs are associated with reduction of costs^62^^,^^63^, thorough studies on return (ROI) and value (VOI) on investment are still missing in the literature.

Unfortunately, the actions implemented by the Brazilian national health system exclusively target children and adolescents, while the economically active population (19-59 years old) is neglected. The available services are still substandard^64^, the advances brought in by the Smiling Brazil program notwithstanding, as was previously discussed. Actions to improve the quality of life of workers as concerns their oral health may be enhanced by means public health policies focusing on prevention, including continuing education, extending the operating hours of services beyond the working time of workers, implementing measures already established in legislation, such as removal of bureaucratic barriers, and reducing costs to employers to promote general and oral health care. Incentives may be afforded to private organizations to participate in health education programs, provide dental care to employees or even better, to establish facilities on their premises^26^^,^^61^.

## CONCLUSIONS

Health and work are integral and intertwined aspects of human development. The relevance of oral health is not dissociated from general and occupational health, and as such it must be enhanced and duly promoted by organizations and society at large. The social and economic costs of the currently high incidence of oral diseases and orofacial pain among Brazilians of economically active age are considerable as a function of the direct impact of these conditions on productivity, not so much due to absenteeism, but to presenteeism. This is to say, workers do not tend to miss work days, but their performance is impaired, and they become susceptible to more serious health problems in the future.

Private dental care is expensive, while public services are inadequate, limited and slow. As a rule, only large, and also some medium-sized companies provide some form of dental care to employees, mostly through outsourced management unrelated to SEOMSS. Such individual approach hinders the formulation of collective strategies to implement oral health promotion and prevention actions in the workplace, and its overall financial weight cannot be properly determined.

Investing in effective and comprehensive public policies and private sector actions in occupational oral health will improve productivity, reduce absenteeism and presenteeism and enhance the quality of life of workers. Continuing education in health as a whole, including oral health, and comprehensive, effective and periodic dental care, preferentially in the workplace, are essential strategies to accomplish these goals.
